# Increased exhalation of hydrogen peroxide in healthy subjects following cigarette consumption

**DOI:** 10.1590/S1516-31802000000400004

**Published:** 2000-07-07

**Authors:** Sandra Baltazar Guatura, José Antônio Baddini Martinez, Patricia Cincotto dos Santos Bueno, Manuel Lopes dos Santos

**Keywords:** Hydrogen peroxide, Smoking, Free radicals, Peróxido de hidrogênio, Tabagismo, Radicais livres

## Abstract

**CONTEXT:**

Increased hydrogen peroxide has been described in the expired breath condensate (H_2_O_2_-E) of several lung conditions, such as acute respiratory distress syndrome, chronic obstructive pulmonary disease and asthma. This technique has been advocated as being a simple method for documenting airway inflammation.

**OBJECTIVE:**

To evaluate H_2_O_2_-E in healthy cigarette smokers, and to determine the acute effects of the consumption of one cigarette on H_2_O_2_-E levels.

**TYPE OF STUDY:**

Prospective, controlled trial.

**SETTING:**

A pulmonary function laboratory in a University Hospital.

**PARTICIPANTS:**

Two groups of healthy volunteers: individuals who had never smoked (NS; n=10; 4 men; age = 30.6 ± 6.2 years) and current cigarette smokers (S; n=12; 7 men; age = 38.7 ± 9.8). None of the volunteers had respiratory symptoms and all showed normal spirometric tests.

**INTERVENTION:**

Expiredair was collected from all volunteers through a face mask and a plastic collecting system leading into a flask with dry ice and pure ethanol. Samples from the group S were collected twice, before and half an hour after the combustion of one cigarette.

**MAIN MEASUREMENTS:**

Expired hydrogen peroxide using the Gallati and Pracht method.

**RESULTS:**

The S and NS groups showed comparable levels of H_2_O_2_- E at basal conditions [NS = 0.74 µM (DP 0.24) vs. S = 0.75 µM (DP 0.31)]. The smokers showed a significant increase in H_2_O_2_-E levels half an hour after the consumption of only one cigarette [0.75 µM (DP 0.31) vs. 0.95 µM (DP 0.22)].

**CONCLUSION:**

The present results are consistent with the concept that smokers increase oxidative stress with elevated production of reactive oxygen species, contributing to the development of smoking-related disorders.

## RESUMO

**CONTEXTO:**

Elevações do peróxido de hidrogênio exalado (H_2_O_2_-E) tem sido descritas em diversas doenças pulmonares tais como a síndrome do desconforto respiratório agudo, doença pulmonar obstrutiva crônica e asma. Essa técnica tem sido preconizada como um método simples capaz de refletir inflamação ao nível das vias aéreas.

**OBJETIVO:**

Avaliaros níveis de H_2_O_2_-E em indivíduos normais e determinar os efeitos agudos do consumo de um cigarro sobre seus valores.

**TIPO DE ESTUDO:**

Ensaio clínico, prospectivo, controlado.

**LOCAL:**

Laboratório de função pulmonar de um Hospital Universitário.

**PARTICIPANTES:**

Dois grupos de voluntários sadios: indivíduos que nunca fumaram [NS; n = 10; 4 homens; idade = 30,6 anos (DP 6,2)] e indivíduos fumantes atuais [S; n = 12; 7 homens; idade = 38,7 anos (DP 9,8)]. Todos os voluntários não apresentavam sintomas respiratórios e exibiam testes espirométricos dentro da normalidade.

**INTERVENÇÃO:**

Ar expirado foi coletado de todos os voluntários utilizando-se uma máscara facial e um sistema colocado em um frasco com gelo seco e etanol absoluto. Amostras do grupo S foram coletadas duas vezes, antes e meia hora após o consumo de um cigarro.

**VARIÁVEIS ESTUDADAS:**

Peróxido de hidrogênio expirado utilizando-se o método de Gallati e Pracht.

**RESULTADOS:**

Ambos os grupos mostraram níveis comparáveis de H_2_O_2_-E em condições basais [NS = 0,74 µM (DP 0,24) vs. S = 0,75 µM (DP 0,31)]. Os fumantes mostraram um aumento significante dos níveis de H_2_O_2_-E meia hora após o consumo de apenas um cigarro [0,75 µM (DP 0,31) vs. 0,95 µM (DP 0,22)].

**CONCLUSÃO:**

Os resultados obtidos estão de acordo com o conceito de que o consumo de cigarros aumenta o estresse oxidativo com produção elevada de espécies reativas do oxigênio (ROS) contribuindo para o desenvolvimento de doenças relacionadas ao tabagismo.

## INTRODUCTION

An imbalance between oxidants and antioxidants has been considered in the pathogenesis of smoking induced lung diseases, such as lung cancer and emphysema. Oxidative stress, which causes an elevation of reactive oxidant species (ROS) may cause a protease-antiprotease imbalance in the lower airways, inducing DNA damage in epithelial cells leading to proteolytic lung injury and carcinogenesis.^1,2^

The increased exposure of smokers’ pulmonary tissues to ROS may result from the inhalation of a large amount of oxidants and free radicals present in cigarette smoke.^3,4^ Another, and possibly more important source of ROS in the lungs of smokers, is the enhanced recruitment of mononuclear phagocytes and polymorphonuclear leukocytes to the lower airways. Activation of these inflammatory cells induces a respiratory burst resulting in marked production of superoxide anion. This oxidant species undergoes spontaneous or enzyme-catalyzed demutation to form hydrogen peroxide (H_2_O_2_).^3,4^ Thus, hydrogen peroxide levels reflect both divalent reduction of oxygen and the demutation of superoxide. Hydrogen peroxide appears to be an important inflammatory mediator itself causing cellular injury and, via further reactions, generating other ROS such as hydroxyl radicals and lipid peroxidation products.^3,4^

Production of H_2_O_2_ has been used in vitro to quantify the respiratory burst of neutrophils.^5^ Different reports have also demonstrated increased hydrogen peroxidein the expired breath condensate (H_2_O_2_-E) of several clinical conditions such as acute respiratory distress syndrome (ARDS), chronic obstructive pulmonary disease (COPD) and asthma.^6-10^ This noninvasive technique has been advocated as a simple method for documenting airway inflammation. Therefore, the present study was designed to evaluate H_2_O_2_-E in healthy cigarette smokers, and to determine the acute effects of the consumption of one cigarette on H_2_O_2_-E levels.

## METHODS

After Hospital Ethical Committee approval, two groups of subjects were studied: those who had never smoked (NS, n = 10) and current cigarette smokers (S, n = 12). None of the individuals had a history of chronic pulmonary disorders or signs of upper and lower respiratory tract infections in the two months prior to collection of the samples. All subjects showed normal values in spirometric tests, and there were no differences between the groups regarding these spirometric parameters (Tables 1 and 2). The S group was significantly older than the NS group [mean age in years, 30.6 (SD 6.2) vs. 38.7 (SD 9.8); P<0.05). The average degree of total smoking exposure for group S was 16.2 (SD 8.6) pack-years. The mean number of cigarettes consumed by group S at the time of the study was 14.3 (SD 5.2) cigarettes per day (Table 2).

**Table 1:** Characteristics of the never-smoker group

Subject	Gender*	Age (years)	FVC+ (%pred.)	FEV_1_δ (%pred.)	FEV_1_/FVC (%pred.)
1	M	23	98.1	94.0	95.1
2	F	39	126.0	113.9	90.6
3	M	26	114.7	120.1	104.4
4	F	26	94.2	97.8	103.1
5	F	26	95.4	98.9	102.7
6	F	37	128.3	126.4	98.2
7	F	39	108.0	94.8	87.8
8	M	27	115.5	105.1	92.4
9	M	28	107.4	112.9	105.6
10	F	35	108.9	105.9	97.5
**Mean (SD)**	**-**	**30.6 (6.2)**	**109.7 (11.8)**	**106.9 (11.1)**	**97.7 (6.2)**

* M: male, F: female; +FVC: forced vital capacity; δFEV_1_: forced expiratory volume in the first second.

**Table 2:** Characteristics of the smoker group

Subject	Gender*	Age(years)	Present smoking (cigarettes/day)	Total smoking (pack-years)	FVC+ (%pred.)	FEV_1_δ (%pred.)	FEV_1_/FVC (%pred.)
1	M	33	4	1.25	102.3	101.0	99.4
2	F	31	10	14	124.1	117.1	95.0
3	M	32	15	13.5	120.5	118.1	100.6
4	F	31	12	8	116.2	118.4	101.5
5	M	45	20	35	102.5	96.8	94.3
6	F	66	10	10	101.3	99.3	97.3
7	M	35	15	18	115.4	112.5	98.0
8	F	41	20	20	114.8	96.8	85.8
9	F	36	15	18	124.7	114.6	95.0
10	M	42	20	25	99.2	90.7	91.4
11	M	34	20	19	118.9	83.3	73.0
12	M	38	10	12.5	101.2	87.0	86.7
**Mean (SD)**	**-**	**38.7 (9.8)**	**14.3 (5.21)**	**16.2 (8.61)**	**111.8 (9.7)**	**103.0 (12.7)**	**93.2 (8.1)**

* M: male, F: female; +FVC: forced vital capacity; δFEV_1_: forced expiratory volume in the first second.

All the expired breath samples were collected in the morning. The smokers were requested to refrain from smoking after 10 P.M. Initially, pulmonary function data were obtained using a VitaTrace spirometer, model VT 130 SL. Subsequently, the participants were asked to breath through a face mask with a one-way valve. The expired air was conducted through a plastic tube connected to a sterile collecting system, leading into a flask containing dry ice and pure ethanol. In this way, approximately 2 ml of breath condensate were collected within 30 minutes of tidal breathing. The samples were immediately stored in a freezer at –80 °C. After the initial sampling, the subjects of the S group were asked to smoke one commercially available cigarette of a popular brand. Half an hour after the end of this cigarette a second collection of expired breath condensate was made.

The measurements of H_2_O_2_ were all made in the same way, using the method described by Gallati and Pracht.^11^ In brief, 100 µl of 420 µM 3,3¢,5,5¢-tetramethylbenzidine (dissolved in 0.42 M citrate buffer, pH 3.8) and 10 µl of 52.5 U/µl of horseradish peroxidase type II (HRP, Sigma Chemicals) were added to 100 µl of the condensate. The reaction proceeded for 20 minutes at room temperature. Subsequently, the mixture was acidified to a pH of 1 with 10 µl of 18 N sulfuric acid. The reaction product was measured spectrophotometrically at 450 nm using an automated microplate reader (Flow/ICN Biomedicals, model Titertek Multiskan MCC340). The absorbance at 450 nm is directly proportional to the concentration of H_2_O_2_. All samples were measured in duplicate and the mean values were used for analysis.

### Statistical methods

The spirometry measurements were expressed as percentages of the predicted values for normal populations using classic equations.^12,13^ All of the results are shown as mean and standard deviation. Statistical comparisons between groups of pulmonary function data and basal H_2_O_2_-E levels were done using Student's t test. Statistical comparisons of H_2_O_2_-E levels in the S group, before and after smoking, were obtained using a paired t test. Pearson coefficients were performed to evaluate correlation between the H_2_O_2_-E levels and cigarette smoking status. Statistical significance was assumed when P < 0.05.

## RESULTS

**Figure 1:**
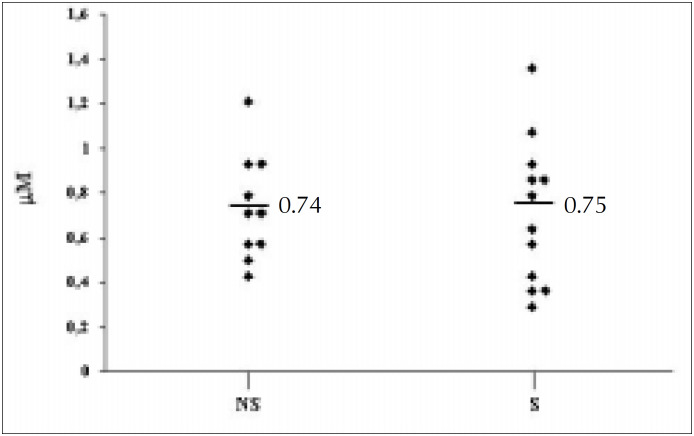
H_2_O_2_-E levels for the group S and NS in basal conditions.

Hydrogen peroxide was detected in the expiratory breath condensate of all those who had never smoked at concentrations ranging between 0.43 and 1.21 µM. The mean and standard deviation of the H_2_O_2_-E values for the NS group were 0.74 µM (SD 0.24) (Figure 1). Hydrogen peroxide was also detected in the breath condensate of all of group S under basal conditions, at levels ranging between 0.29 and 1.07 µM. The mean and standard deviation of the H_2_O_2_-E values for the S group were 0.75 µM (SD 0.31). Statistical analysis showed no significant differences between group S and group NS H_2_O_2_-E basal levels (Figure 1). In addition, there were no significant correlations found between initial H_2_O_2_-E levels and the total or present smoking history for group S subjects (r = 0.125 and r = 0.057, respectively).

**Figure 2:**
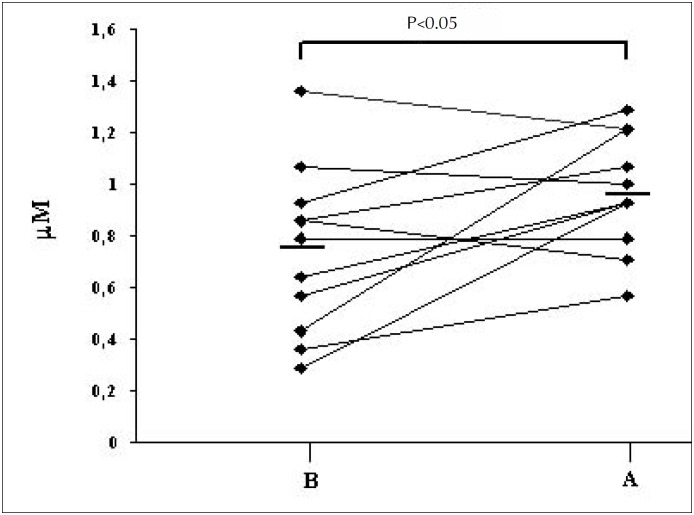
H_2_O_2_-E levels for the group S before (B) and after (A) patients had smoked one cigarette.

Group S showed a mean H_2_O_2_-E value of 0.95µM (SD 0.22) for samples collected half an hour after the subjects had smoked a regular cigarette. The H_2_O_2_-E levels post-smoking [0.95 µM (SD 0.22)] were significantly higher than the basal values [0.75 µM (SD 0.31)] (Figure 2).

## DISCUSSION

Investigations of chemical compounds in the exhaled air should be scrutinized by respiratory biology researchers because they may lead to the development of methods for evaluating inflammation of the lung parenchyma and airways. For example, several studies have demonstrated measurable amounts of nitric oxide in human expired gas, both under physiological and pathological conditions, and this observation has attracted growing interest in this field.^14-16^

The first description of hydrogen peroxide in the expired air of humans was in 1983.^17^ Since then high H_2_O_2_-E levels have been detected under a number of different conditions such as ARDS, asthma and COPD.^6-10^ Although such results strongly suggest a role for expired hydrogen peroxide as a marker for respiratory tract inflammation, the number of investigations and publications in the area remains small.

Recently a study by Horvath et al.^18^ evaluated the relationship between nitric oxide and hydrogen peroxide in the exhaled air of asthmatic patients and also evaluated bronchial hyperreactivity, pulmonary function tests, and cellular counts of induced sputum. These results suggested for the first time that H_2_O_2_-E might be a more sensitive and reliable marker of airway inflammation than exhaled nitric oxide in this population.

Smoking is the most important risk factor for COPD and lung cancer, and it is believed by some that the generation of ROS in the lower airways may substantially contribute to the pathogenesis of these dis-orders.^2,3^ In such conditions, the detection of high levels of H_2_O_2_-E in smokers with no clinical or functional evidence of disease, would add credibility to the hypothesis that H_2_O_2_ contributes significantly to the pathogenic mechanisms of smoking-related diseases. In addition, this type of measurement could become a useful clinical tool, able to detect subjects with smoking-induced pulmonary inflammation at its very earliest stages.

In the present study hydrogen peroxide was detected in the expired breath condensate of all NS subjects. In fact, the measured levels were well above the values previously described for non-smoking volunteers in the literature.^9,10^ These differences could result from contamination of breath condensate with saliva that contains high amounts of hydrogen peroxide. In this study we used a whole facemask and, since the volunteers were required to breathe nasally, the chances of significant saliva contamination is quite small. Such findings could also be explained by the type of equipment we have employed for collecting condensate. Most of the researchers in the field have previously collected expired condensate through endotracheal tubes or via oral breathing. Using a facemask and nasal breathing, we have collected condensate that could reflect the metabolism of lung parenchyma and upper airways as well. In such a situation the upper airways and paranasal sinus could be a significant source of H_2_O_2_-E, in a similar way to that already described for nitric oxide.^19^ A third important possibility for explaining these differences is related to the quality of the air in São Paulo city. Air pollution is still a health concern in this big city and its effects on the H_2_O_2_-E levels are at present unknown. The S group also had a significantly higher mean age than the NS group. As the influences of aging on H_2_O_2_-E levels are presently unknown, this difference may be a potential source of bias in our results. Further studies are necessary to clarify such aspects.

Group S showed a significant increase in the mean H_2_O_2_-E level, half an hour after the consumption of only one cigarette (Figure 2). In part, this finding may be due to inhalation of H_2_O_2_ originating from the cigarette combustion and possibly still present in the airways at the time of the condensate collection. This is unlikely since the thirty minutes rest period should have been enough to dissipate most of the inhaled hydrogen peroxide accumulated in the airways from cigarette combustion alone. Cigarette smoke has about 5 × 10^14^ free radicals per inhalation which, in contact with the wet surfaces of the respiratory epithelium, can generate superoxide radicals and H_2_O_2_.^3,4^ In addition, such smoke-generated radicals may damage cellular membranes in the respiratory tract leading to lipid peroxidation and additional oxidative stress.^2,3,4^ Therefore, the present increases in H_2_O_2_-E levels thirty minutes after cigarette consumption probably reflect acute, smoking-induced, paranasal, upper and lower airway and lung parenchyma injuries. Another contributory factor in these findings may be the acute influx and sequestration of inflammatory cells in the lung parenchyma. It has been shown that cigarette smoking can acutely promote pulmonary vascular retention of marked neutrophils.^20^ The mechanisms related to this phenomenon are not completely understood, but may involve activation of adhesion molecules, changes in leukocyte cytoskeletons, and local hemodynamic disorders.^20-22^ Whatever the mechanisms involved, the acute elevation of H_2_O_2_-E levels found in this study reflect ROS respiratory overloading secondary to the smoking habit.

The S and NS groups showed comparable levels of H_2_O_2_-E under basal conditions (Figure 1). This suggests that smoking leads to transient changes in the H_2_O_2_-E, which returns to normal levels in healthy subjects after some hours of abstinence. Our results disagree with a recent paper which reported a fivefold increase in basal H_2_O_2_-E for a group of 33 cigarette smokers in comparison to 27 non-smokers.^23^ Although the authors had included in their investigation only subjects without respiratory symptoms and with a normal physical examination, pulmonary function tests were not performed. Therefore we cannot rule out the possibility that some of the smoking individuals in that study had asymptomatic COPD.

Dekhuijzen et al.^9^ have shown that increased H_2_O_2_-E occurs in subjects with stable COPD and even more so in patients with an exacerbation of COPD. Our data shows at least the occurrence of transient elevations of H_2_O_2_-E after challenging healthy individuals by cigarette smoking. Therefore we can hypothesize that there may be an initial phase in the evolution of COPD, when high levels of H_2_O_2_-E are continuously found in subjects without significant respiratory complaints. This event may happen in COPD-susceptible smokers for whom an oxidant load would repeatedly overcome the antioxidant protection in the lower airways. In this scenario the measurement of H_2_O_2_-E could become a sensitive and non-invasive method for showing enhanced generation of ROS, and may be used to detect subjects with a higher risk for developing smoking-related pulmonary diseases. Larger and more complete studies will need to be performed to confirm this hypothesis.

## CONCLUSION

Increased H_2_O_2_-E occurs in healthy subjects after the consumption of only one cigarette. Although such elevation could just be a marker of smoking, this result is consistent with the concept that, in smokers, elevated production of ROS increases oxidative stress, which may contribute to the development of smoking-related diseases.
